# Toward the Role of EFL/ESL Students’ Silence as a Facilitative Element in Their Success

**DOI:** 10.3389/fpsyg.2021.737123

**Published:** 2021-08-16

**Authors:** Jing Hu

**Affiliations:** School of College English Teaching and Research, Henan University, Kaifeng, China

**Keywords:** student silence, facilitative function, EFL/ESL students, success, English language learning

## Abstract

Following the recent evolution of research perspectives toward student silence, an increasing number of studies have sought to empirically probe into the beneficial role of this variable in students’ success. Yet, a limited number of review studies have been carried out to illustrate the complex nature of student silence and its positive consequences (e.g., success, increased learning outcomes, etc.). Hence, this study aims to review different definitions of “student silence” to elucidate its facilitative function in EFL/ESL students’ success. Providing empirical evidence, the role of student silence as a facilitative element in English language learning was proved. Finally, some pedagogical implications for EFL/ESL teachers and teacher trainers are also discussed.

## Introduction

For decades, students’ silence in English as a second/foreign language (ESL/EFL) classes had been perceived as a detrimental learning behavior, inhibiting interactions between teachers and students (Wang and Liu, 2021). However, in recent years, several scholars (e.g., Hanh, 2020; Harumi, 2020; Peng, 2020; Tsui and Imafuku, 2020) have suggested that silence may not always be an impediment for students and may rather serve a facilitative function in language learning. In this regard, Harumi and King (2020) have emphasized the need to distinguish the pedagogical roles of silence as a “voluntary productive communicative resource” or a “mode of learning” and of reticence as “withdrawal from learning” (p. 6). In an attempt to characterize the pedagogical role of student silence, Hanh (2020), stated that silence may be beneficial for students’ success as it offers more opportunities for reflection and cognition. In the same vein, Harumi (2020) also noted that students’ silence on the surface has the potential to promote L2 learning by leaving more space for “attentive listening, thinking, and reformulating ideas” (p. 39).

The notion of silence is literally defined as “the absence of vocalization” (Bosacki, 2005, p. 6). For many previous studies (e.g., Granger, 2004; Liu, 2005, 2006; Liu and Jackson, 2009; Delima, 2012), the concept of student silence had negative connotations. In his study, Granger (2004), for instance, referred to students’ silence as “disobedience,” “conflict,” and “misbehavior.” Similarly, Liu and Jackson (2009) also defined student silence as an emotional reaction against the teachers’ authority and a means of passively expressing unfavorable feelings. However, with the latest evolution of research perspectives toward student silence, an increasing number of recent studies has adopted positive perspectives to define this concept. According to some studies (e.g., Hanh, 2020; Tsui and Imafuku, 2020), students’ silence should not be deemed as an absence of thought or absence of communication but rather it should be considered as another means of communication.

Given the significance of classroom silence in second language learning, several empirical studies have been carried out to investigate the role of this factor in English language learning (e.g., Bista, 2012; Bao, 2014; Banks, 2016; Min, 2016; King and Smith, 2017; Juniati et al., 2018; King et al., 2020; Maher and King, 2020; Hongboontri et al., 2021). However, most of the researchers have chosen to probe into the impeditive role of students’ silence in their learning. Furthermore, a small number of studies in the form of review have been conducted to explain the multidimensional nature of student silence. Hence, in the current review article, the researcher endeavored to illustrate the concept of student silence, on the one hand, and to elaborate on its facilitative effects on EFL/ESL students’ success, on the other hand.

### The Concept of Student Silence

Student silence is an elusive and ambiguous concept with various communicative connotations that cannot be easily characterized. As such, there is a wide range of controversy regarding the definition of this concept. To be more specific, Granger (2004) simply defined student silence as “the mere absence of speech” (p. 3), as opposed to Liu (2002), who characterized this concept as a communicative strategy through which students show respect to their teachers and classmates. As another example, Bruneau (2008) conceptualized student silence as “a lack of communication contact with other classroom participants” (p. 78), whereas Meyer (2009) referred to this concept as another means of communication. Two distinctive attitudes were applied in conceptualizing the notion of student silence. Those who had a negative viewpoint toward student silence characterized it as students’ unresponsiveness, inattentiveness, and disengagement in educational contexts (Nakane, 2007; Ping, 2010; Choi, 2015). On the other hand, those who had positive perceptions about students’ silence defined this concept as “a voluntary productive communicative resource able to enhance L2 learning opportunities” (Harumi and King, 2020, p. 6).

In an attempt to categorize different types of student silence, Kurzon (2007) classified this variable into two groups, namely *intentional silence* and *unintentional silence*. While students’ intentional silence is strategic and deliberately employed for certain reasons, their unintentional silence is accidental and unconscious, occurring when a student is extremely anxious or ashamed.

### The Facilitative Role of EFL/ESL Students’ Silence in Their Success

Historically, in almost all educational settings, students’ verbal behavior has attracted more favorable attention than their silence. More specifically, in second language learning contexts, students’ speech as the language output has been the focus of much research. However, considering visible/audible behaviors as the sole language output indicates an extremely simplistic position toward students’ advancement (Innocenti, 2002; Galletly and Bao, 2015). In this regard, Bao (2020) noted that based on how silence is deployed, “the occurrence of inner speech in the learner’s system deserves to be considered as a type of production, especially when ideas or thoughts are taking shape in the mind” (p. 18). Based on this logic, the place of student silence in EFL/ESL classes has been revisited. That is, several scholars (e.g., Harumi, 2020; Harumi and King, 2020; Karas and Faez, 2020; King et al., 2020; Peng, 2020; Tsui and Imafuku, 2020) re-examined the role of student silence in second language learning. Harumi and King (2020), for instance, referred to student silence as a “mode of learning” that enables students to listen more attentively, which in turn enhances their academic success. Similarly, Harumi (2020) also explained that students’ silence as a learning tool can offer more space for students’ reflection, resulting in increased learning outcomes. Additionally, King et al. (2020) also suggested that students’ intentional silence can give them an invaluable chance to revise and reformulate their ideas, which may help them to overcome their feeling of anxiety. With a low level of anxiety, students are more likely to engage in classroom activities (Liu and Jackson, 2011; King, 2014; Effiong, 2016).

## Empirical Studies

The positive and facilitative role of student silence in English language learning was empirically proved by several recent studies (e.g., Hanh, 2020; Harumi, 2020; Humphries et al., 2020; King et al., 2020; Maher, 2020; Maher and King, 2020; Peng, 2020). For instance, Harumi (2020) investigated classroom silence, its consequences, and different appropriate approaches to interact with this phenomenon. To do this, 56 English language teachers took part in this study. The required data were gathered through observations and open-ended questionnaires. Analyzing the obtained data, the researcher found that students’ silence can serve a facilitative function in their success if language teachers know how to interact with it. In another study, Hanh (2020) studied the silent behavior of 85 EFL students who voluntarily participated in the study. Analyzing students’ responses to the questionnaire and interview questions, the researcher evinced what factors contribute to students’ silence and how the silent behavior of students can promote their learning. Besides, to this end, King et al. (2020) probed EFL students’ silence in relation to their sense of anxiety. In so doing, structured classroom observations, self-report reflection sheets, and stimulated recall interviews were employed to collect data. In light of the results of analyses, the researchers explained in what ways students’ intentional silence can reduce their sense of anxiety.

## Conclusion and Pedagogical Implications

In this review article, the concept of student silence, its distinctive definitions, its different types (i.e., intentional, unintentional), and its positive consequences (i.e., success, increased learning outcomes) were explained. Furthermore, some empirical studies conducted on student silence were also summarized to prove the positive effects of students’ silence on their learning outcomes. According to what was theoretically and empirically reviewed, it can reasonably be inferred that the silent behavior of students can play a pivotal role in their academic success only if it is appropriately utilized and managed by classroom participants (i.e., teachers, students). The implications emerging from this review relate specifically to EFL/ESL teachers. First and foremost, teachers should not perceive students’ intentional silence as their academic disengagement since they typically employ silence to think, reflect, and listen more attentively (Harumi and King, 2020). Second, teachers should not push their students to talk as it may increase their sense of fear and anxiety (King, 2014; King and Smith, 2017). Besides, the findings of this review have some pedagogical implications for teacher trainers. Given the significance of the silent behavior of students in their success (Maher, 2020; Maher and King, 2020; Peng, 2020; Tsui and Imafuku, 2020), teacher trainers should alter teachers’ attitudes toward this phenomenon and instruct them how to appropriately interact with their students’ silence.

## Author Contributions

JH composed this review independently and revised it before it was submitted.

## Conflict of Interest

The author declares that the research was conducted in the absence of any commercial or financial relationships that could be construed as a potential conflict of interest.

## Publisher’s Note

All claims expressed in this article are solely those of the authors and do not necessarily represent those of their affiliated organizations, or those of the publisher, the editors and the reviewers. Any product that may be evaluated in this article, or claim that may be made by its manufacturer, is not guaranteed or endorsed by the publisher.
